# The Chromatin Remodeler Chd8 Regulates Hematopoietic Stem and Progenitor Cell Survival and Differentiation During Zebrafish Embryogenesis

**DOI:** 10.3390/ijms262110805

**Published:** 2025-11-06

**Authors:** Abrar Ahmed, Xiaona Wei, Dan Zhong, Rahat Ullah, Wei Li, Lili Jing

**Affiliations:** 1 Engineering Research Center of Cell & Therapeutic Antibody, Ministry of Education, School of Pharmacy, Shanghai Jiao Tong University, Shanghai 200240, China; ahmedchd22@gmail.com (A.A.); weixiaona@sjtu.edu.cn (X.W.); rahat_2020@sjtu.edu.cn (R.U.); 2 Joint International Research Laboratory of Biomaterials and Biotechnology in Organ Repair (Ministry of Education), School of Life Science, Shanghai University, Shanghai 200444, China; mzd_98@163.com; 3 Institute of Cardiovascular Sciences, Shanghai Engineering Research Center of Organ Repair, School of Life Science, Shanghai University, Shanghai 200444, China; 4 Core Facility and Technical Service Center, School of Pharmacy, Shanghai Jiao Tong University, Shanghai 200240, China

**Keywords:** zebrafish, chromodomain helicase DNA-binding protein 8 (CHD8), hematopoietic stem and progenitor cells (HSPCs), p53, Brd4

## Abstract

Chromodomain helicase DNA-binding protein 8 (CHD8), a frequently mutated gene in autism spectrum disorder (ASD), is an ATP-dependent chromatin remodeler with emerging roles in hematopoiesis. While CHD8 is known to maintain hematopoietic stem and progenitor cells (HSPCs) in the bone marrow, its function during developmental hematopoiesis remains undefined. Here, using a zebrafish model, we demonstrate that *chd8* loss severely depletes the HSPC pool in the caudal hematopoietic tissue through a p53-dependent apoptotic mechanism. Furthermore, *chd8*^−/−^ embryos exhibit a p53-independent expansion of myelopoiesis. *chd8* deficiency upregulates *brd4*, which promotes systemic inflammatory cytokine expression. Inhibiting *brd4* alleviates cytokine expression, suppresses excessive myelopoiesis, and restores HSPC development. Our findings reveal a dual regulatory mechanism in which *chd8* governs HSPC development by repressing p53-mediated apoptosis and constraining *brd4*-driven immune cell differentiation.

## 1. Introduction

Hematopoietic stem and progenitor cells (HSPCs) are the foundation of the vertebrate hematopoietic system, responsible for the lifelong production and maintenance of all blood cell lineages [1]. HSPCs first arise during embryogenesis from the dorsal aorta in the aorta–gonad–mesonephros (AGM) region. Following their emergence, HSPCs migrate to the fetal liver in mammals or the caudal hematopoietic tissue (CHT) in zebrafish before ultimately colonizing the mammalian bone marrow or zebrafish kidney, where they sustain adult hematopoiesis [2,3,4].

In the fetal liver or CHT, HSPCs undergo rapid expansion and differentiation. The extensive proliferation induces replicative stress, which can trigger apoptosis to eliminate compromised cells and preserve the integrity of the HSPC pool [5,6,7,8]. Conversely, concurrent survival signals are essential to maintain a balance between cell death and propagation, ensuring proper HSPC expansion and preventing excessive depletion [9]. This critical equilibrium between apoptosis and survival, and between expansion and differentiation, is tightly regulated by elaborate signaling pathways, including transcriptional and epigenetic mechanisms [5].

Chromatin remodeling enzymes are essential regulators of chromatin structure and function, playing critical roles in stem cell biology. Among these, the chromodomain helicase DNA-binding (CHD) family is a major subtype defined by the presence of two chromodomains, a central helicase/ATPase domain, and a DNA-binding domain [10]. These proteins utilize energy from ATP hydrolysis to modulate DNA accessibility for transcription factors and other epigenetic modifiers, making them indispensable for proper developmental processes [11]. Notably, several CHD proteins, including CHD2, CHD4, and CHD7, have been established as key regulators of stem cell maintenance and differentiation [12,13,14]. 

CHD8 was originally identified as one of the most frequently mutated genes in autism spectrum disorders (ASDs) [15,16]. Prior research has largely focused on its critical role in brain development, including regulating pluripotency and early neurodifferentiation [17,18]. Recent studies have revealed its functions in hematopoiesis. CHD8 governs HSPC survival and stemness by restricting p53 activity in the adult bone marrow [19,20], promotes erythroid differentiation [21] and regulates Treg cells [22]. Although CHD8 is expressed throughout development, its role in HSPC development during embryogenesis remains unknown because of the early embryonic lethality of *Chd8*-null mice [23].

We previously demonstrated that a *chd8* mutation in zebrafish disrupted the intestinal epithelial integrity, leading to a microbiota imbalance that impairs HSPC development [24]. But whether *chd8* intrinsically affects HSPC survival remains unknown. In this study, we found that *chd8* is dispensable for the initial emergence and migration of HSPCs, but it is essential for their subsequent survival within the CHT by suppressing p53-dependent apoptosis. We also found that *chd8* loss leads to p53-independent myeloid differentiation, and that Brd4 inhibition rebalances HSPC development and myeloid differentiation.

## 2. Results

### 2.1. Chd8 Is Expressed in Developing HSPCs and chd8^−/−^ Zebrafish Exhibit Significant Lethality

**Figure 1:**
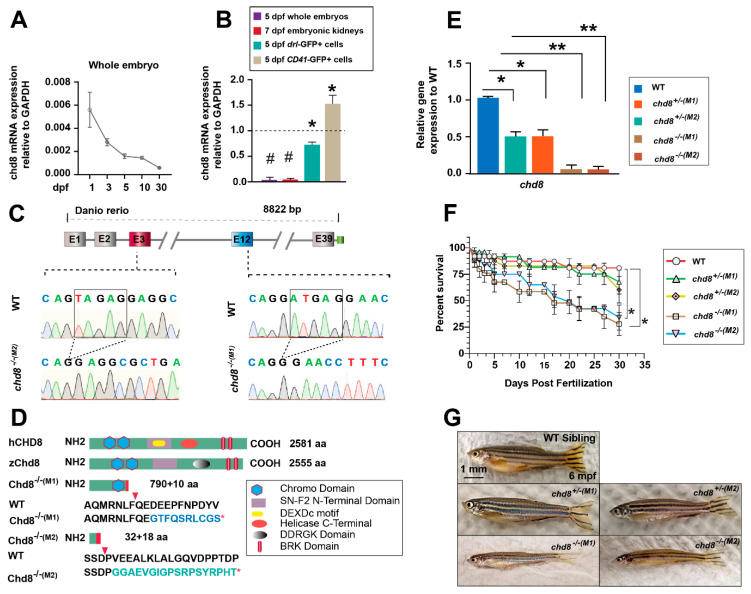
Characterization of *chd8* mRNA expression and two *chd8* mutant lines. (**A**) Developmental expression profile of *chd8* in whole embryos. Transcript levels were quantified by RT-qPCR and normalized to *gapdh*. (**B**) *chd8* expression in whole embryos, embryonic kidneys, sorted *drl*-GFP^+^-hematopoietic cells, and *CD41*-GFP^+^-HSPCs. (**C**) Upper panel: genomic structure of zebrafish *chd8*, with targeted exons 3 and 12 highlighted. Lower panel: Sanger sequencing chromatograms confirming the deletion in *chd8*^−/−^ mutants. The deleted nucleotides are shown in squares. (**D**) Domain architecture of human CHD8, zebrafish Chd8, and the truncated mutants (M1, M2) predicted from the genomic deletions. The asterisk indicates the premature stop codon. (**E**) *chd8* transcript levels in WT, *chd8*^+/−^, and *chd8*^−/−^ embryos from two mutant lines. (**F**) Survival analysis of WT, *chd8^+/−^*, and *chd8*^−/−^ zebrafish from 1 dpf to 1 mpf (n = 100–200 per group). (**G**) Representative images of adult WT and *chd8*^+/−^, and *chd8*^−/−^ (6 mpf). Data are presented as mean ± SD from three independent experiments (two-tailed Student’s *t*-test, * *p* < 0.05, ** *p* < 0.01, ^#^ *p* < 0.001). WT, wild type; dpf/mpf, days/months post-fertilization; bp, base pairs; aa, amino acids.

To study the intrinsic role of *chd8* in hematopoiesis, we first analyzed its expression at different developmental stages and within hematopoietic cells using real-time quantitative PCR (RT-qPCR). *Chd8* exhibited a dynamic expression profile, with transcript levels peaking during early developmental stages and gradually declining as embryogenesis proceeded (Figure 1A). Furthermore, *chd8* expression is highly enriched in fluorescence-activated cell sorting (FACS)-purified *drl*-GFP^+^ hematopoietic cells [25] and *CD41*-GFP^+^ HSPCs [8] compared to the whole embryos. This enrichment was also observed in embryonic kidneys, the site of definitive hematopoiesis in zebrafish (Figure 1B). The specific enrichment of *chd8* in HSPC-containing tissues and populations supports its potential for a cell-intrinsic function in these cells.

To study the hematopoietic development, we used two zebrafish *chd8*^−/−^ mutant lines previously established in our laboratory, *chd8*^M1−/−^ and *chd8^M2−/−^* [24]. The zebrafish Chd8 gene and the mammalian CHD8 protein are highly conserved. The amino acid homology between zebrafish Chd8 and human CHD8 is approximately 70%. The full-length zebrafish Chd8 protein comprises 2555 amino acids, and the two lines produce truncated proteins of 800 aa and 50 aa, respectively, due to premature termination codons (Figure 1C,D). RT-qPCR analysis showed a ~50% reduction in *chd8* mRNA in heterozygotes (*chd8*^+/−^) and nearly undetectable levels in homozygous mutants (*chd8*^−/−^) (Figure 1E). Both mutant lines exhibited significant mortality rates, reaching 35–45% by 5 days post-fertilization (dpf), with a continued increase thereafter (Figure 1F). A small fraction of mutants survived to adulthood but were notably leaner than their wild-type (WT) counterparts at 6 months post-fertilization (mpf) (Figure 1G). *chd8* heterozygotes showed no significant mortality or morphological abnormalities (Figure 1E,G). The viable *chd8* mutant embryos give us a useful tool to study embryonic hematopoiesis. The two mutant lines exhibited similar hematological phenotypes. Unless specifically mentioned, the subsequent results are primarily from the *chd8^M1−/−^,* referred to hereafter as *chd8*^−/−^ for simplicity.

### 2.2. Loss of chd8 Does Not Affect Primitive Hematopoiesis

**Figure 2:**
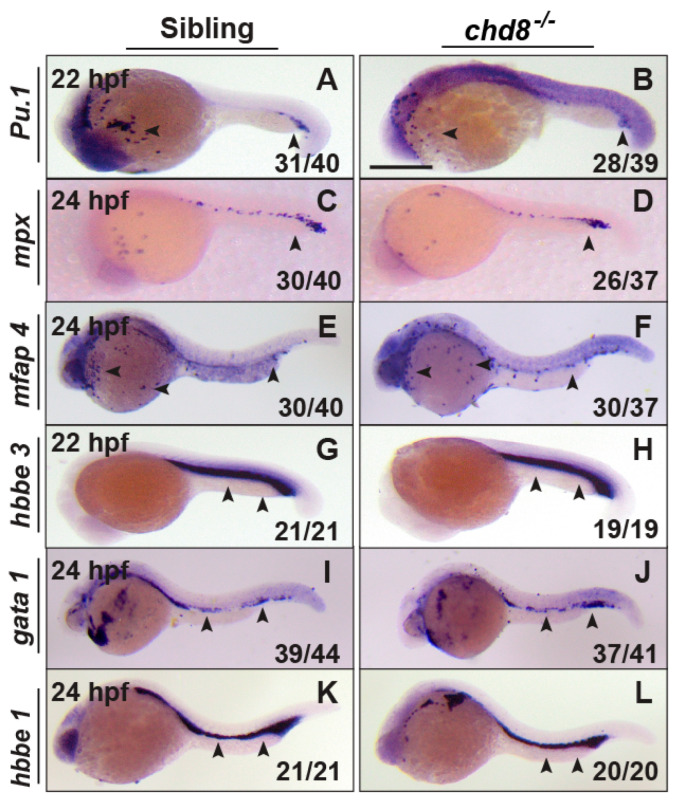
*Chd8* loss does not affect primitive hematopoiesis. WISH analysis of *PU.1* (**A**,**B**), *mpx* (**C**,**D**), *mfap4* (**E**,**F**), *hbbe3* (**G**,**H**), *gata 1* (**I**,**J**), and *hbbe1* (**K**,**L**) in *chd8*^−/−^ and their corresponding WT sibling embryos at 22–24 hpf. The fraction (n/n) in the bottom right corner indicates the number of embryos displaying the representative phenotype over the total number examined. WISH, whole-mount in situ hybridization; Scale bar, 200 µm.

Similar to mammals, zebrafish hematopoiesis occurs in two consecutive waves: primitive and definitive [4]. We first investigated whether *chd8* mutants exhibited defects in primitive hematopoiesis. Using whole-mount in situ hybridization (WISH), we found that the expression of myeloid markers *Pu.1* (at 22 hpf), *mpx* and *mfap4* (at 24 hpf) showed no difference between *chd8*^−/−^ mutants and their WT siblings. Similarly, the expression of erythroid progenitor marker *gata 1* (at 24 hpf), and the erythrocyte markers *hbbe3* and *hbbe1* (at 22–24 hpf) were unaltered in *chd8*^−/−^ embryos (Figure 2). These results suggest that primitive myeloid and erythroid development remains intact in the absence of *chd8*, indicating that *chd8* is not required for primitive hematopoiesis.

### 2.3. Loss of chd8 Impairs HSPC Development in the CHT

**Figure 3:**
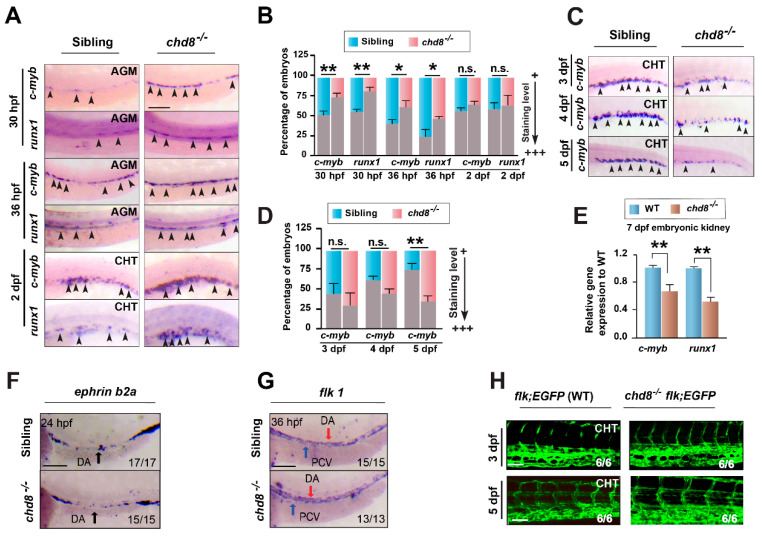
*chd8* is required for maintaining HSPCs in the CHT. (**A**,**C**) Representative WISH images tracking definitive HSPC markers *c-myb* and *runx1* in WT sibling and *chd8*^−/−^ embryos from 30 hpf to 5 dpf. (**B**,**D**) Quantification of the WISH signal for *c-myb* and *runx1* in (**A**,**C**), respectively. (**E**) RT-qPCR analysis of *c-myb* and *runx1* expression in the embryonic kidney at 7 dpf. (**F**,**G**) WISH of the arterial marker *ephrinb2a* at 24 hpf and the vascular marker *flk1* at 36 hpf. (**H**) Live imaging of vascular plexus in the CHT region of Tg (*flk*: GFP) WT and *chd8*^−/−^ embryos at 3 and 5 dpf. Data in (**B**,**D**,**E**) are presented as mean ± SD from three independent experiments (two-tailed Student’s *t*-test, n = 20–30 embryos per group, each experiment, * *p* < 0.05, ** *p* < 0.01, n.s., not significant). AGM, aorta–gonad–mesonephros; CHT, caudal hematopoietic tissue. Scale bars, 100 µm (**A**,**F**,**G**); 50 µm (**H**).

To characterize the role of *chd8* in definitive hematopoiesis, we analyzed the expression of HSPC markers *cmyb* and *runx1*. At 30 and 36 hpf, the expression of *c-myb* and *runx1* in the AGM was properly specified and slightly elevated in *chd8*^−/−^ mutants (Figure 3A,B), indicating HSPC emergence is normal or potentially enhanced. At 2 dpf, *cmyb* and *runx1* expression remained intact, suggesting successful HSPC migration to the CHT. However, by 3 dpf, *cmyb* expression in the CHT was decreased, and by 5 dpf, it was strongly reduced in *chd8*^−/−^ mutants (Figure 3C,D). This reduction persisted in the embryonic kidneys at 7 dpf (Figure 3E). Taken together, these results revealed that while HSPC initiation and migration were unaffected in *chd8*^−/−^ mutants, their transient expansion within the CHT is severely impaired. We also examined heterozygous mutants and observed no hematopoietic defects, indicating that a single allele loss of *chd8* does not impair hematopoiesis in zebrafish (Figure S1).

Since HSPCs originate from arterial vessels and their development is influenced by vasculogenesis and blood flow [26], we assessed vascular integrity. The expression of arterial (*ephrinb2*) and endothelial (*flk1*) markers was normal at 24 and 36 hpf (Figure 3F,G). Live imaging of the vascular plexus in Tg(*flk1:EGFP*) [5] embryos at 3 and 5 dpf further confirmed the normal vascular morphology in *chd8*^−/−^ mutants (Figure 3H). We also did not find any circulation defects in the mutants. These findings together support a specific effect of *chd8* in definitive hematopoiesis.

### 2.4. Loss of chd8 Leads to Enhanced Immune Cell Differentiation

**Figure 4:**
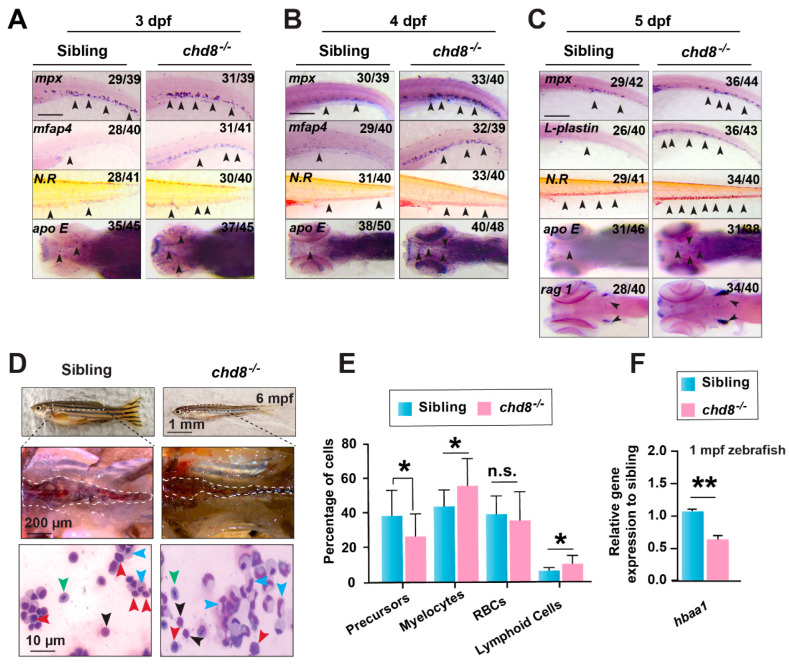
*chd8* loss leads to excessive myeloid and lymphoid differentiation. (**A**–**C**) WISH for hematopoietic lineage markers in WT sibling and *chd8*^−/−^ embryos from 3 to 5 dpf. Neutrophils were labeled with *mpx* and *l-plastin,* and macrophages were labeled with *mfap4*, *apoE*, and neutral red (N.R.) staining; lymphoid cells were labeled with *rag1*. (**D**) Kidney morphology and May-Grünwald-Giemsa-stained WKM cells from 6-month-old WT and *chd8*^−/−^ zebrafish. Cell types are indicated by arrows: blue, myelocytes; black, lymphoid cells; red, precursors; green, red blood cells. (**E**) Quantification of different cell populations in the WKM, based on morphological analysis in (**D**). (**F**) RT-qPCR analysis of hemoglobin gene *hbaa1* expression in 1-month-old WT and *chd8*^−/−^ zebrafish. Data are presented as mean ± SD from three independent experiments (two-tailed Student’s *t*-test, n = 5–6 fish per experiment, * *p* < 0.05, ** *p* < 0.01, n.s., not significant). WKM, whole kidney marrow. Scale bars, 100 µm (**A**–**C**).

To explore the impact of *chd8* on HSPC function, we analyzed a series of lineage markers in *chd8*^−/−^ embryos. WISH revealed a significant upregulation of the neutrophil markers *mpx* and *L-plastin* at 3, 4 and 5 dpf in *chd8*^−/−^ embryos (Figure 4A–C). Similarly, expression of the macrophage markers *mfap4*, *apoE,* and neutral red-stained macrophages was increased in the *chd8*^−/−^ CHT. We also observed elevated expression of the lymphoid marker *rag1*. These findings indicate that *chd8* deficiency enhances both myeloid and lymphoid differentiation. As with HSPC, *chd8* haploinsufficiency did not produce detectable abnormal phenotypes in differentiation (Figure S1).

We next asked whether the increased differentiation potential persists into adulthood. May-Grünwald-Giemsa staining of whole kidney marrow (WKM) from 6-month-old fish showed that, consistent with the embryonic phenotype, precursor cells were reduced while mature lymphoid and myeloid populations were expanded in *chd8*^−/−^ (Figure 4D,E). Tu et al. reported that Chd8 loss causes anemia in mice [21], and we observed an increase in the erythroid marker *hbbe1* and benzidine-stained globin in *chd8^−/−^* (Figure S2). But we found a significant reduction in *hbaa1* expression starting from 1-month-old mutants (Figure 4F), suggesting a potential late-onset erythroid defect. These hematopoietic phenotypes were recapitulated in a second mutant allele, *chd8*^M2−/−^ (Figure S3). Thus, our results demonstrate that *chd8* loss disrupts HSPCs development and lineage output.

### 2.5. Loss of chd8 Induces p53-Mediated Apoptosis in HSPCs

**Figure 5:**
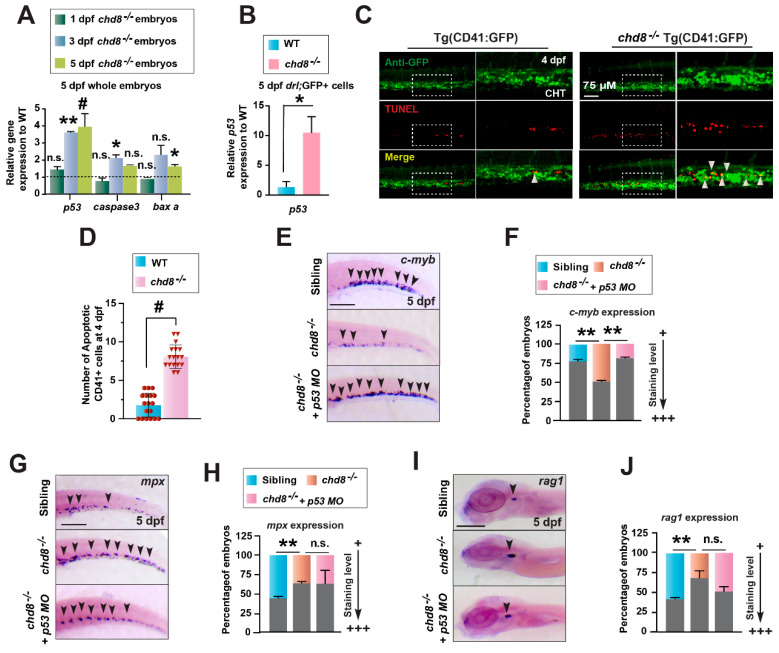
HSPCs undergo *p53*-dependent apoptosis in *chd8*^−/−^ embryos. (**A**) RT-qPCR analysis of *p53, caspase3, and baxa* expression in WT and *chd8*^−/−^ embryos at 1, 3, and 5 dpf. (**B**) RT-qPCR analysis of *p53* in *drl*-GFP^+^ cells sorted from WT and *chd8*^−/−^ embryos. (**C**) Representative images of *CD41*-GFP^+^ and TUNEL double staining in WT and *chd8*^−/−^ embryos at 4 dpf. The white arrowheads indicate apoptotic HSPCs in the CHT. (**D**) Quantification of TUNEL-positive HSPCs from the images in (**C**) (n = 6 embryos per group). (**E**,**G**,**I**) WISH analysis for *c-myb*, *mpx*, *rag1* in WT and *chd8*^−/−^ embryos following injection with *p53* MO. (**F**,**H**,**J**) Quantitative analysis of the WISH results in (**E**,**G**,**I**), respectively. Data are presented as mean ± SD from three independent experiments (two-tailed Student’s *t*-test, n ≈ 10–20 embryos per group, each experiment, * *p* < 0.05, ** *p* < 0.01, ^#^ *p* < 0.001, n.s., not significant). MO, morpholino. Scale bars: 100 µm (**E**,**G**,**I**).

Previous studies have shown that *chd8* safeguards HSPCs by suppressing p53-dependent apoptosis in the bone marrow [22]. To determine if a similar mechanism operates in zebrafish, we assessed apoptosis in *chd8*-deficient embryos. Quantitative analysis revealed a significant upregulation of key apoptosis-related genes, including *p53*, *caspase-3*, and *baxa*, in whole embryos at 1, 3, and 5 dpf (Figure 5A). This elevation was even more pronounced within sorted *drl*^-^GFP^+^ hematopoietic cells at 5 dpf (Figure 5B), suggesting hematopoietic cell-intrinsic activation of the apoptotic program. Consistent with this, TUNEL assays demonstrated a substantial increase in apoptotic *CD41*^-^GFP^+^ HSPCs in the CHT of *chd8*^−/−^ embryos (Figure 5C,D). These findings imply that the definitive hematopoiesis defect in *chd8*^−/−^ originates from increased HSPC apoptosis. To directly test whether p53 upregulation mediates this phenotype, we knocked down p53 in *chd8*^−/−^ embryos using a morpholino (MO) [27]. WISH showed a complete rescue of *cmyb*^+^ HSPCs in both the CHT and the embryonic kidney of *chd8*^−/−^ embryos (Figure 5E,F), confirming that the HSPC loss is p53-dependent. Interestingly, *p53* knockdown failed to suppress the enhanced myelopoiesis in *chd8*^−/−^ embryos (Figure 5G,H), and did not inhibit increased erythroid differentiation (Figure S2D,E). In contrast, it partially rescued *rag1* expression (Figure 5I,J). These results suggest the enhanced myeloid and erythroid differentiation in *chd8*^−/−^ embryos occurs largely via a p53-independent mechanism. This finding aligns with the work of Tu et al., indicating that Chd8 possesses p53-independent functions in murine erythroid development. Collectively, our data demonstrate that *chd8* deficiency depletes HSPCs by triggering p53-dependent apoptosis, but inhibiting p53 restores survival without fully rescuing their lineage differentiation defects.

### 2.6. BET Inhibitor PFI-1 Restores HSPC Production and Differentiation

**Figure 6:**
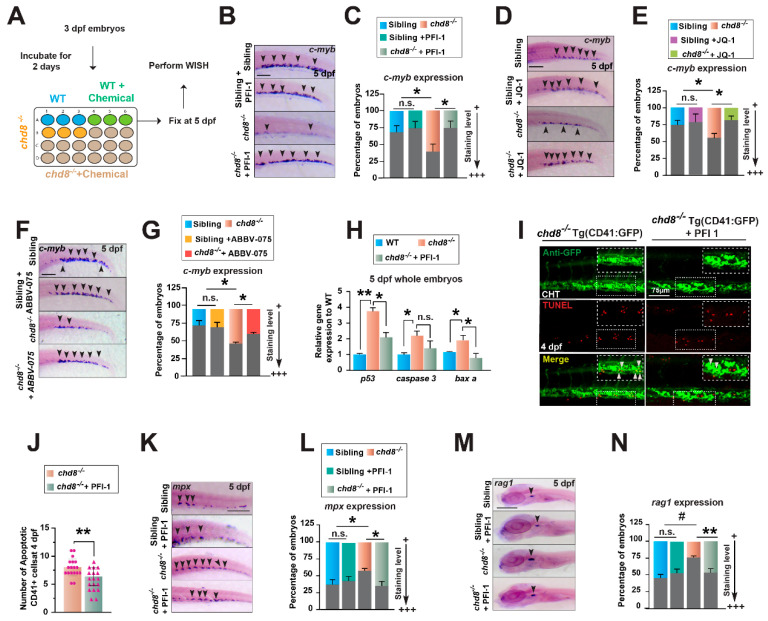
The compound PFI-1 restores HSPC survival and differentiation. (**A**) Schematic of the chemical treatment regimen. Embryos were exposed to compounds from 3 to 5 dpf and subsequently analyzed by WISH. (**B**,**D**,**F**) WISH analysis of *c-myb* at 5 dpf in WT and *chd8*^−/−^ embryos treated with DMSO or the BET inhibitors PFI-1 (25 µM), JQ-1 (0.5 µM), and ABBV-075 (0.05 µM). (**C**,**E**,**G**) Quantification of WISH results in (**B**,**D**,**F**). (**H**) RT-qPCR analysis of *p53*, *caspase3,* and *baxa* in WT and chd8^−/−^ embryos treated with DMSO or PFI-1. (**I**) Representative images of CD41-GFP and TUNEL double staining in *chd8*^−/−^ embryos at 4 dpf treated with DMSO or PFI-1. Apoptotic HSPCs are indicated by white arrowheads in the CHT. (**J**) Quantification of the apoptotic HSPCs in (**I**). (K, M) WISH for *mpx* and *rag1* at 5 dpf in WT siblings and *chd8*^−/−^ embryos treated with DMSO or PFI-1. (**L**,**N**) Quantification of WISH results in (**K**,**M**). Data are presented as mean ± SD from three independent experiments (two-tailed Student’s *t*-test, n ≈ 20 embryos per group in each experiment, * *p* < 0.05, ** *p* < 0.01, ^#^ *p* < 0.001, n.s., not significant). Scale bars, 100 µm (**B**,**D**,**F**,**K**,**M**).

To identify additional regulators in *chd8*-mediated hematopoiesis, we performed a rescue screen with the Cayman Chemical Epigenetics Screening Library, based on the known role of *chd8* in epigenetic regulation. Embryos from *chd8*^+/−^ intercrosses were treated with library compounds (n = 96) from 3 to 5 dpf and assessed for *c-myb* expression at 5 dpf (Figure 6A). Several compounds restored *c-myb* expression in *chd8*^−/−^, with the BET (Bromodomain and Extra-Terminal) proteins inhibitor PFI-1 [28] exhibiting the strongest rescue effect (Figure 6B,C). BET proteins are transcriptional regulators that mainly bind to acetylated histones to control gene expression in fundamental processes such as cell proliferation, differentiation, and apoptosis [29]. To further validate their role, we employed additional BET inhibitors, JQ-1 and ABBV-075 [29]. Treatment with these compounds also significantly restored *c-myb*^+^-HSPC populations in *chd8*^−/−^ embryos (Figure 6D–G). We next investigated whether BET inhibition influences HSPC survival. In *chd8*^−/−^ embryos, PFI-1 treatment significantly downregulated the expression of *p53*, *caspase-3,* and *baxa* (Figure 6H). Consistently, the TUNEL assay demonstrated a marked reduction in apoptotic CD41-GFP+-HSPCs in the CHT following PFI-1 treatment (Figure 6I,J), indicating that PFI-I antagonizes p53-mediated apoptosis to rescue HSPC survival. Impotently, PFI-1 treatment also significantly reduced the enhanced expression of *mpx* and *rag1* in *chd8*^−/−^ mutant embryos at 5 dpf, suggesting it also counteracts the aberrant lineage differentiation driven by *chd8* loss. Notably, these drug doses did not affect hematopoiesis in WT siblings (Figure 6B–G,K–N), indicating a specific corrective effect in the mutant context. Thus, BET inhibition restores HSPC development by suppressing p53-mediated apoptosis and normalizing excessive immune cell differentiation in the *chd8*^−/−^ embryos.

### 2.7. PFI-1 Targets Brd4 in chd8^−/−^ to Restore HSPC Production and Differentiation

**Figure 7:**
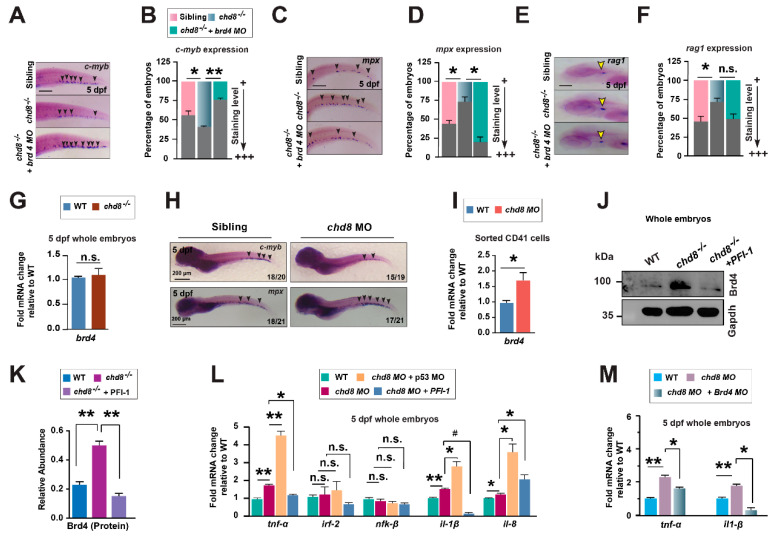
*brd4* knockdown rescues HSPCs in *chd8*^−/−^ embryos. (**A**,**C**,**E**) WISH analysis for *c-myb*, *mpx*, and *rag1* at 5 dpf in WT siblings, and *chd8*^−/−^ embryos with and without *brd4* morpholino injection. (**B**,**D**,**F**) Quantification of WISH analysis in (**A**,**C**,**E**). (**G**) RT-qPCR analysis of *brd4* expression in WT and *chd8*^−/−^ whole embryos. (**H**) WISH analysis for *c-myb*, *mpx* in WT siblings and embryos injected with *chd8* MO. n/n in the lower right corner shows the number of embryos with similar staining patterns/total number of embryos examined. (**I**) RT-qPCR analysis of *brd4* expression in sorted CD41-GFP^+^ cells from WT and *chd8* morphant embryos (5 dpf). (**J**) Western blot analysis for Brd4 protein in WT and *chd8*^−/−^ embryos treated with DMSO or PFI-1. GAPDH is shown as a loading control. (**K**) Quantification of Brd4 protein in (**J**). (**L**) RT-qPCR analysis of cytokine expression in WT and *chd8* morphant embryos treated with DMSO or PFI-1 or injected with *p53* MO. (**M**) RT-qPCR analysis of cytokine expression in WT and *chd8* morphant embryos injected with *brd4* MO. Data are expressed as mean ± SD from three independent experiments (two-tailed Student’s *t*-test, n ≈ 20 embryos per group, each experiment, * *p* < 0.05, ** *p* < 0.01, *^#^ p* < 0.001, n.s., not significant). ng, nanogram. Scale bars, 100 µm (**A**,**C**,**E**).

We next sought to identify the potential protein target through which PFI-1 exerts its rescue effect in *chd8*^−/−^. PFI-1 is a potent BET inhibitor, particularly blocking the activity of BRD2 and BRD4 [28]. We first characterized the expression of their zebrafish homologs, *brd2a*, *brd2b,* and *brd4*, and confirmed their presence in hematopoietic cells (Figure S4A,B). To determine which of these is functionally required for the phenotype, we performed MO-mediated knockdown of each gene. While knockdown of *brd2a*, *brd2b* or a combination of both failed to restore HSPCs in *chd8*^−/−^ mutants (Figure S5A–F), knockdown of *brd4* completely rescued HSPC production and suppressed the enhanced myeloid differentiation (Figure 7A–D). Furthermore, *brd4* inhibition also partially restored lymphoid differentiation (Figure 7E,F). Moreover, simultaneous knockdown of *brd2a* and *brd2b* in the *brd4* morphant background did not augment this rescue (Figure S5G–L), indicating that *brd4* is the primary target of PFI-1 in *chd8*^−/−^.

Next, we examined the relationship between Chd8 and Brd4. *brd4* mRNA levels was unchanged in the whole *chd8*^−/−^ embryos (Figure 7G). To test if *chd8* regulates *brd4* in HSPCs, we knocked down *chd8* in Tg(CD41:GFP) embryos using a MO [30], which recapitulated the *chd8*^−/−^ mutant phenotypes (Figure 7H). This knockdown significantly increased *brd4* expression in CD41-GFP^+^ HSPCs (Figure 7I). Furthermore, Brd4 protein was strongly upregulated in the whole *chd8*^−/−^ embryos (Figure 7J,K), suggesting potential post-transcriptional dysregulation. The fact that *chd8* loss causes *brd4* upregulation, while *brd4* knockdown rescues the hematopoietic defects in *chd8*^−/−^ mutants, positions *brd4* as a critical downstream effector of *chd8*. Therefore, we conclude that Chd8 mediates definitive hematopoiesis through at least two parallel pathways: by repressing p53-dependent apoptosis and by constraining Brd4 activity.

Finally, we previously demonstrated that the microbiota dysbiosis in *chd8*^−/−^ drives elevated cytokine expression, which contributes to the host hematopoietic defects [24]. As Brd4 is known to regulate inflammatory gene expression, we asked whether Brd4 participates in the inflammatory response. Both PFI-1 treatment and *brd4* knockdown successfully attenuated the systemic cytokine expression in *chd8*^−/−^ mutants, whereas *p53* knockdown did not produce this effect (Figure 7L,M). Therefore, Brd4 inhibition restores normal hematopoiesis partially through mitigating the pro-inflammatory cytokine expression.

## 3. Discussion

Recent studies have shown that CHD8 functions as a key regulator of HSPC survival and stemness in the bone marrow via suppressing p53 activity [19,20]. However, its role in HSPC development during embryogenesis remains unknown. Our study reveals that the zebrafish *chd8*^−/−^ embryos retained primitive hematopoiesis but showed severe defects in definitive hematopoiesis in the CHT. *chd8* is essential for HSPC survival by suppressing p53-dependent apoptosis. Furthermore, *chd8* loss leads to enhanced myeloid differentiation through a p53-independent mechanism. Inhibiting *brd4* attenuated myeloid differentiation and promoted HSPC development in *chd8*^−/−^ partially via decreasing elevated inflammatory cytokine expression. Our results demonstrate that *chd8* plays critical and multidimensional regulatory roles in HSPC development.

Definitive HSPCs transiently emerge from the AGM before migrating to the CHT. Chd8 does not regulate early HSPC emergence but is critical for their survival and development in the CHT from 3 dpf. Nascent HSPCs seldom proliferate in the AGM region but enter an active in cell cycle and undergo rapid division in the CHT [7,31]. Such extensive cell division in a short timeframe poses a risk of replicative stress, potentially activating the DNA replication checkpoint and inducing p53-dependent apoptosis [7,32]. We hypothesize that *chd8* functions to suppress unwanted apoptosis in proliferating HSPCs, providing a critical window for DNA damage repair and ensuring the successful expansion of the HSPC pool. Previous studies have shown that dysregulation of alternative splicing activates p53-dependent apoptosis in HSPCs [33,34]. Meanwhile, Wdr5-mediated H3K4 methylation is crucial for HSPC survival in the CHT by maintaining genomic stability [7]. Our study identified another essential regulator involved in this process. In *chd8*^−/−^, both primitive hematopoiesis and vascular systems develop normally. And *chd8* mRNA is highly enriched in *CD41*-GFP^+^ HSPCs. These data support that *chd8* likely maintains HSPC survival through intrinsic mechanisms. Future studies, including the use of p53 mutants, are needed to formally prove that this function is cell autonomous. Furthermore, in the mouse bone marrow, CHD8 deficiency activates ATM kinase to stabilize P53 protein and CHD8 also directly binds to P53 to regulate its transactivation [20]. How Chd8 suppresses p53 activation during zebrafish development remains unknown. Future omics studies, such as those utilizing activator-targeted chromatin accessibility sequencing (ATAC-seq), will reveal the chromatin accessibility regulated by Chd8, thereby helping elucidate the mechanism by which it suppresses p53 activation.

In addition to HSPC defects, *chd8*^−/−^ zebrafish embryos displayed enhanced immune cell differentiation that persisted into adulthood. *Chd8*^−/−^ mice die between embryonic days 5.5 and E7.5 (E5.5-E7.5) [23]. Using the Mx-Cre inducible system, deletion of *Chd8* in murine hematopoietic cells causes a significant reduction in HSPCs and lineage differentiation [19,20]. This discrepancy may be largely attributed to the distinction between a constitutive systemic knockout in zebrafish and a hematopoietic-specific deletion in mice. The phenotype in *chd8*^−/−^ zebrafish likely involves both cell-autonomous and non-autonomous mechanisms. Chd8 may function cell-intrinsically to maintain HSPC survival. We previously showed that *chd8* mutants harbor an imbalanced microbiota that drives elevated inflammatory cytokines (e.g., *tnfa* and *il1b*) in the host, which in turn non-cell-autonomously impairs HSPC differentiation and production [24]. In this study, we found that BET inhibitors and Brd4 knockdown reduced systemic cytokine production and restored balanced HSPC development in *chd8*^−/−^. In contrast, *p53* knockdown did not affect cytokine production nor restore differentiation, but specifically rescued HSPC survival. This supports that enhanced myelopoiesis is related to elevated cytokines. Moreover, enhanced differentiation persisted in germ-free (GF) *chd8*^−/−^ embryos [24], indicating that additional, microbiota-independent, genetic mechanisms promote differentiation. Previous studies showed that *Chd8* directly regulates pluripotency and early neural differentiation through chromatin structure in mouse embryonic stem cells (ESCs) and neural progenitor cells (NPCs) [17,18]. We downloaded the RNA-seq data from mouse HSPC (WT vs *Chd8*^−/−^) [22], and found that despite no apparent enhancement in differentiation, myeloid and lymphoid differentiation and inflammatory pathways were significantly upregulated in mouse *chd8*^−/−^ HSCs (Figure S6). This suggests that *Chd8* may intrinsically negatively regulate these pathways in HSPCs, possibly through chromatin compaction, thereby contributing to precise differentiation control. The differentiation defects in *chd8*^−/−^ zebrafish are likely the collective effects of HSPC-intrinsic regulation and extrinsic signaling. Future studies using tissue-specific knockdown and rescue experiments will help elucidate the cell-autonomous and non-autonomous functions of Chd8 in HSPC development. However, given that CHD8 is a high-risk gene for ASD—a condition often associated with immune dysregulation [35,36]—and its mutations are constitutive, the viable *chd8*^−/−^ zebrafish larvae provide a powerful tool for analyzing hematopoietic defects caused by CHD8 deficiency at the organismal level.

PFI-1 is a highly selective BET inhibitor that particularly targets BRD2 and BRD4 by blocking their ability to bind to acetylated histone tails—a binding crucial for gene transcription [28]. Our study revealed that in *chd8*^−/−^ embryos, PFI-1 primarily targets Brd4 to alleviate hematopoietic defects. As a key epigenetic regulator, BRD4 cooperates with various master transcription factors to control gene expression [29] and is known to positively regulate inflammatory genes such as IL1B [37,38]. Knocking down Brd4 may directly repress these inflammatory cytosines, thereby restoring the developmental balance of HSPCs. We also found that Brd4 inhibition partially rescues HSPC apoptosis in *chd8*^−/−^, likely by mitigating the pro-apoptotic effects induced by high levels of inflammatory cytokines such as IL1B and TNFα [39,40]. Indeed, we found that MO-mediated inhibition of *il1b* in *chd8*^−/−^ partially reduced the expression of apoptosis-related gene (Figure S6). In addition, *brd4* is known to directly promote granulopoiesis in zebrafish [41]. Thus, upregulation of Brd4 may drive myeloid differentiation directly. The exact function of Brd4 during HSPC development requires further study. Although *brd4* expression remained unchanged in whole embryos, it was significantly upregulated in CD41^+^-HSPCs. Thus, *chd8* may mediate *brd4* expression within HSPCs. Notably, the increase in Brd4 protein levels in *chd8* mutants far exceeded that of its mRNA levels, implying that Chd8 may regulate Brd4 protein synthesis or stability through post-transcriptional mechanisms. The cell type-specific regulation of Brd4 by Chd8 and the underlying regulatory mechanisms require further investigation. This study reveals that Chd8 restricts Brd4 activity during embryogenesis.

In conclusion, our findings demonstrate that Chd8 maintains HSPC development by suppressing p53-dependent activity. Furthermore, Chd8 loss drives p53-independent enhanced myeloid differentiation, and Brd4 inhibition restores normal myeloid differentiation and supports HSPC development. Our studies elucidate the critical roles of Chd8 in regulating developmental hematopoiesis.

## 4. Materials and Methods

### 4.1. Zebrafish Maintenance and Embryo Handling

Zebrafish (Danio rerio) strains, including wild-type AB and transgenic lines Tg(*CD41*: GFP), Tg(*drl*: GFP), and Tg(*flk*: GFP), were raised and maintained with a 14 h light/10 h dark cycle in a circulating water system at 28.5 °C, conductance 450–550 μS, and pH 7.0 ± 0.5 [42]. WT Siblings and mutant embryos were collected at the desired stage and grown in an E3 medium (5 mM NaCl, 0.17 mM KCl, 0.33 mM CaCl_2_, 0.33 mM MgSO_4_) with a density of 80–120 embryos per 10 cm Petri dish. Embryos were staged by hours post-fertilization (hpf) and days post-fertilization (dpf). Zebrafish were maintained, handled, and bred according to standard protocols from the Institutional Animal Care and Use Committee of Shanghai Jiao Tong University, China.

### 4.2. Genotyping of Mutant Lines

The *chd8^−/−(M1)^* and *chd8^−/−(M2)^* mutant lines were generate by CRISPR/Cas9 technology as previously described [24]. For genotyping, DNA fragments of 388 bp and 420 bp (flanking the respective *chd8* target sites) were amplified by polymerase chain reaction (PCR) from genomic DNA. Mutants were identified using the T7 endonuclease I assay (NEB, M0302S) and confirmed by Sanger sequencing. The primers used to amplify the *chd8* target sequences are listed in Table 1.

### 4.3. Whole Mount In Situ Hybridization

Whole-mount in situ hybridization (WISH) was performed on 4% paraformaldehyde (PFA) -fixed zebrafish embryos. The anti-sense probes (*c-my*, *pu.1*, *runx1*, *hbbe1* and *hbbe3*, *mpx*, *mfap4*, and *rag1*) were synthesized by in vitro transcription using SP6 or T7 polymerase (Ambion) with Digoxigenin RNA Labelling Mix (Roche, Mannheim, Germany). The WISH procedure was carried out as previously described [43]. Stained embryos were mounted in 100% glycerol and imaged using an Olympus SZX16 stereomicroscope or a BX53 microscope (Olympus, Tokyo, Japan).

### 4.4. Neutral Red, Benzidine, and Sudan Black Staining

To minimize pigmentation and enhance staining signal, embryos were treated with 1-phenyl 2-thiourea (PTU) until the desired stages. For macrophage visualization, live 4–6 dpf embryos were incubated in the dark for 6–8 h at 28.5 °C with 2.5 µg/mL Neutral Red (A600652, Sangon Biotech, Shanghai, China) in E3 medium [44]. For hemoglobin detection, live embryos were incubated in the dark for 30 min in a benzidine solution, prepared by combining 2 mL of 5 mg/mL benzidine (B108444, Aladdin, CA, USA) in methanol, 16.7 µL of 3 M sodium acetate, 100 µL of H_2_O_2_, and 2.483 mL of H_2_O [45]. Following incubation, embryos were washed with PBS containing 0.1% Tween 20 (PBT) and fixed overnight in 4% paraformaldehyde (PFA).

For Sudan Black staining, embryos at the desired stages were fixed overnight in 4% methanol-free formaldehyde (Polysciences), rinsed three times with PBS, and incubated in Sudan black B (SB; Sigma-Aldrich, Saint-Quentin Fallavier, France) solution at room temperature for 1 h [46]. Embryos were thoroughly washed with 70% ethanol and rehydrated with PBS and 0.1% Tween 20 (PBT). To better visualize the staining, embryos were incubated in a bleach solution (10% KOH, 30% H_2_O_2_, 10% Tween 20) for 5 min and then washed with PBT. Images of the stained embryos were taken through an SZX16 stereomicroscope or a BX53 microscope (Olympus, Tokyo, Japan).

### 4.5. Morpholinos

The antisense morpholinos (MOs) were purchased from Gene Tools and resuspended in nuclease-free water to a 1 mM stock solution. Embryos were injected at the 1–4 cell stage with 1–7 ng/nL of MO into the yolk. Injected embryos were raised to 5 dpf for subsequent staining. The MO sequence is provided in Table 2.

### 4.6. Chemical Screen

A screen of epigenetic small molecules (Cayman Chemicals, 11076) was performed at concentrations of 6, 12.5, and 25 µM, using DMSO as a vehicle control. WT and *chd8*^−/−^ mutant embryos at 3 dpf were arrayed into 24-well plates (15 embryos per well in 600 µL of E3 medium). Plates were wrapped in aluminum foil to protect light-sensitive compounds and incubated at 28.5 °C until 5 dpf. The chemicals were then washed out 3 times with E3 medium. The toxicity of the chemicals was determined, and healthy embryos at 5 dpf were then fixed in 4% PFA at 4 °C overnight (O/N) for WISH. The additional BET inhibitors were obtained commercially: PFI-1 (Lot# QLDMMEO), (+)-JQ1 (Med Chem Express, Cat# HY-13030/CS-0581), and ABBV-075 (Mivebresib).

### 4.7. May–Grünwald–Giemsa Staining of Adult Whole Kidney Marrow Cells

Adult zebrafish of the desired genotype were euthanized in 0.4% tricaine. The kidney was then dissected via a midline incision and placed in 0.9× PBS containing 5% FBS. Whole kidney marrow (WKM) cells were isolated by mechanical dissociation through strenuous pipetting in 0.4 mL of the same buffer, followed by filtration through a 40-µm cell strainer (Falcon, New York, NY, USA). The resulting cell suspension was centrifuged onto glass slides using a cytocentrifuge (Sigma-Aldrich) at 400 rpm for 4 min. Slides were stained with May-Grünwald and Giemsa (Sigma-Aldrich) for morphological analysis and differential cell counting.

### 4.8. Gene Expression by Real-Time qPCR

Total RNA was isolated from whole embryos, kidneys, and sorted cells using Trizol reagent (Thermo Fisher Scientific, 10296028, Carlsbad, CA, USA) according to the manufacturer’s instructions. RNA was reverse-transcribed into cDNA using either the PrimeScript RT reagent Kit with gDNA Eraser (Takara, RR047A, Kyoto, Japan) or the Hifair^®^ II 1st Strand cDNA Synthesis SuperMix for qPCR (Yeasen Biotech, 11123ES10, Shanghai, China). Quantitative real-time PCR (qPCR) was performed on a StepOnePlus system (Applied Biosystems, San Francisco, CA, USA) with TB Green Premix Ex Taq II (Takara, RR820A, Japan) or Hieff^®^ qPCR SYBR Green Master Mix (Yeasen Biotech, 11203ES08, Shanghai, China). Gene expression was quantified using the 2^–ΔΔCT^ method. The RT-qPCR primers are listed in Table 3.

### 4.9. Flow Cytometry and Cell Sorting

The *CD41* GFP^+^ and *drl* GFP^+^ cells from WT and *chd8*^−/−^ embryos were sorted out using FACS AriaIII (Becton, Dickinson and Company, Franklin Lakes, NJ, USA). Briefly, embryos of the desired stage were anesthetized in 0.4% tricaine, and single cells were collected by chopping the embryos with a blade. Following that, the chopped embryos were incubated for 20 min at 37 °C in 38 µg/mL Liberase (05401119001, Roche, Basel, Switzerland). The liberase reaction was stopped by adding 10% FBS, followed by filtration through a 40 µm filter and centrifugation (1500 rpm, at 4 °C for 10 min). The supernatant was discarded, and cells were resuspended in PBS containing 1% FBS for subsequent studies.

### 4.10. TUNEL Immunostaining

WT sibling and *chd8*^−/−^/ Tg (*CD41*: GFP) embryos were fixed at 4 dpf in 4% PFA overnight at 4 °C and dehydrated in methanol at −20 °C for 1 h. After serial rehydration, embryos were permeabilized with proteinase K (20 mg/mL) at room temperature (RT) for 40 min. The apoptotic cells were stained with Bright Red Apoptosis Detection Kit (Vazyme Biotech Co., Ltd., Shanghai, China) for 90 min at 37 °C. After washing with 1XPBS, embryos were blocked for 2 h at RT in blocking buffer (PBS + 1% DMSO + 0.1% Triton X-100+ 1% bovine serum albumin). To amplify the GFP signal, embryos were incubated with an anti-GFP antibody (Dia-an, Cat# 2057), followed by Alexa Fluor 488-conjugated goat anti-mouse secondary antibody (Invitrogen, Waltham, MA, USA) incubation. Images were taken through a confocal microscope (Leica, Wetzlar, Germany).

### 4.11. Statistical Analysis

GraphPad Prism 8.0.2 (GraphPad Software, San Diego, CA, USA, 2019) was used to analyze the data. The values of all triplicate experiments are presented as mean ± standard deviation (SD), and significance was determined by a 2-tailed Student t-test. For WISH and other staining results, embryos were divided into strong and weak staining groups. For statistical analysis, only the strongly stained groups of wild-type and mutant/morphant were compared.

## Figures and Tables

**Table 1:** PCR primers used for genotyping.

Mutant Lines	Forward Primer	Reverse Primer
*chd8^−/−(M1)^*	CAAACTTTTTGACAAGATGG	ACCAAGAGAAGAGCTCAGCA
*chd8^−/−(2)^*	TTGCCTCTGTTATAGCCATGA	ACACACACTTTTGCTGGCAA

**Table 2:** List of Morpholino (MO) used.

Target Genes	MO Sequence	Reference
*p53*	GCGCCATTGCTTTGCAAGAATTG	[27]
*chd8*	GAGAATGGAATCATAACTTACTTGA	[30]
*brd2a*	CCACCTGAGACTAAAACAGAGACAA	[47]
*brd2b*	AGACTGGTTGATGGCCGCCTCCATC	[47]
*brd4*	TCATGTCTAATGACACAGAAAGAGA	[47]

**Table 3:** Primers used for RT-qPCR.

Genes	Forward Primer	Reverse Primer
*zp53*	ACCACTGGGACC AAACGTAG	CAGAGTCGCTTCTTCCTTCG
*zbax a*	GGCTATTTCAACCAGGGTTCC	TGCGAATCACCAATGCTGT
*zcaspase 3*	ATGCCAAGCCTCAATCCC	TCACAATGTATCCAAGCTTTCG
*zchd8*	AAGGAGGACAAAGACTAGCAGTG	AAGATGCAGCTGTAGTGGTGG
*zrunx1*	TTTGGGACGCCAAATACG	AAACCCTCGCTCATCTTCC
*zc-myb*	TCGCCAGCTTTCTACCAAA	CAGGGTTGAGGACTTTCTGC
*zhbaa1*	CAAGGCTGTTGTTAAGGC	ATTCTGGCGAGGGCTTC

## Data Availability

The original contributions presented in this study are included in the article/Supplementary Material. Further inquiries can be directed to the corresponding authors.

## Supplementary Materials

The following are available online at https://www.mdpi.com/article/10.3390/ijms262110805/s1.
