# Supplementary material for: The Chromatin Remodeler Chd8 Regulates Hematopoietic Stem and Progenitor Cell Survival and Differentiation During Zebrafish Embryogenesis
Source: Int J Mol Sci. 2025 Nov 6;26(21):10805. doi: 10.3390/ijms262110805 (PMC12609008; doi:10.3390/ijms262110805)
Supplement: Supplementary file 1 [file ijms-26-10805-s001.zip › ijms-3944806-supplementary.pdf]

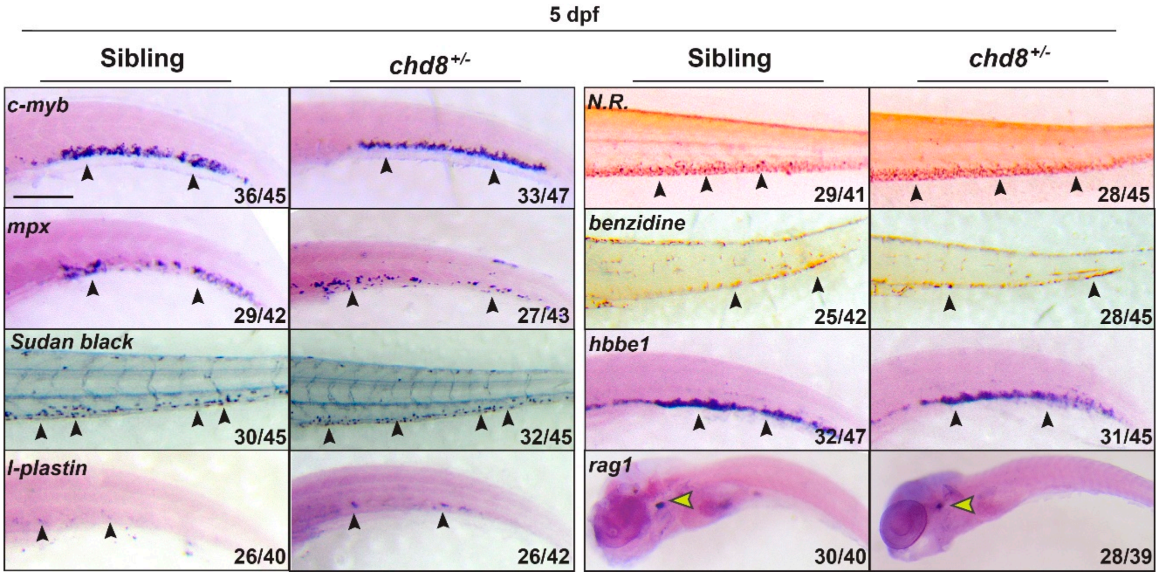

Figure S1. Heterozygosity for *chd8* (*chd8*$^{+/-}$) had no observable effects on hematopoiesis.

WISH for *c-myb*, *mpx*, *l-plastin*, *hbbe1*, *rag1*, and cytochemical staining with Sudan Black, neutral red, and benzidine in WT siblings and *chd8*$^{+/-}$ embryos at 5 dpf. The fraction (n/n) in the bottom right corner indicates the number of embryos with the representative phenotype over the total number examined. dpf, days post-fertilization; WISH, whole-mount in situ hybridization. Scale bar, 100 µm.

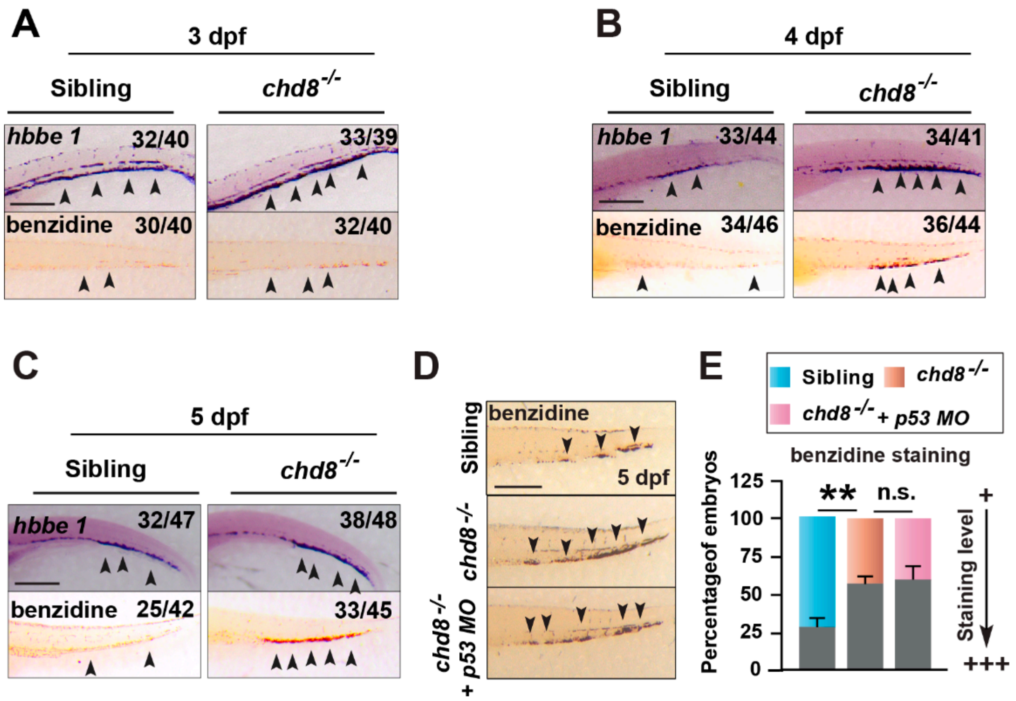

Figure S2. The loss of *chd8* enhanced erythroid differentiation in a *p53*-independent manner.

(A, B, C) WISH for *hbbe1* and benzidine staining in WT siblings and *chd8*$^{-/-}$ embryos at 3, 4, and 5 dpf. The fraction (n/n) in the upper right corner indicates the number of embryos with the representative phenotype over the total number examined. (D) Benzidine staining in WT, *chd8*$^{-/-}$ embryos and *chd8*$^{-/-}$ embryos following injection with *p53* MO. (E) Quantitative analysis of the results in (D). Data are presented as mean ± SD from three independent experiments (two-tailed Student's *t*-test, n≈10-20 embryos per group, each experiment in (D, E) **p < 0.01, $^{\#}$p < 0.001, n.s., not significant). MO, morpholino. Scale bars, 100 µm.

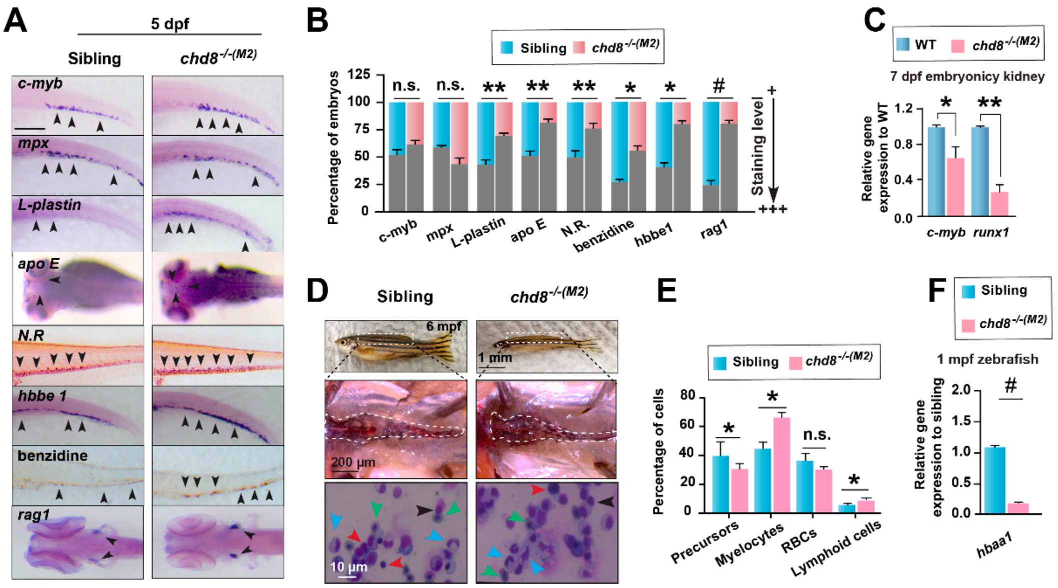

Figure S3. ***chd8*$^{-/-(M2)}$ mutants display similar hematopoietic defetcs as *chd8*$^{-/-(M1)}$.**

(A) WISH analysis of *c-myb, mpx, l-plastin*, *apo E*, *hbbe1*, *rag1,* and cytochemical staining with neutral red and benzidine in WT siblings and *chd8*$^{-/-(M2)}$ embryos at 5 dpf. (B) Quantification of WISH and staining results in (A). (C) RT- qPCR analysis of *c-myb* and *runx1* expression in 7-dpf embryonic kidney in WT and *chd8*$^{-/-(M2)}$ embryos. (D) Morphology of the kidney and May-Grunwald-Giemsa staining of whole kidney marrow (WKM) cells obtained from WT siblings and *chd8*$^{-/-(M2)}$ adult zebrafish (6 mpf). Arrows denote distinct lineages: blue (myelocytes), black (lymphoid cells), red (precursors), green (red blood cells, RBCs). (E) Quantification of the relative abundance of different cell populations in the WKM, based on morphological assessment in (D). (F) RT-qPCR analysis of *hbaa1* expression in 1 mpf zebrafish in both WT siblings and *chd8*$^{-/-(M2)}$. Data are expressed as mean ± SD from three independent experiments (two-tailed Student's *t*-test, n= 15-20 embryos per group, each experiment, $^{*}p < 0.05$, $^{**}p < 0.01$, $^{\#}p < 0.001$, n.s., not significant). mpf, months post-fertilization. Scale bar, 100 μm (A).

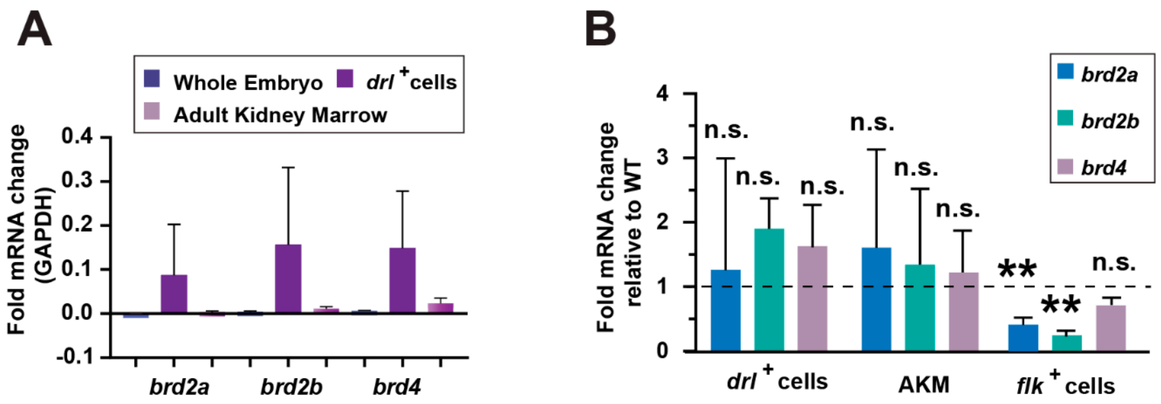

Figure S4. Expression of *brd2a*, *brd2b*, and *brd4* in hematopoietic cells. (A) RT-qPCR analysis of *brd2a, brd2b, and brd4* expression in WT whole embryos, sorted *drl-GFP*$^{+}$-hematopoietic cells from WT embryos, and in adult kidney marrow from 6-month-old WT fish. (B) RT-qPCR analysis of *brd2a, brd2b, and brd4* expression in sorted *drl-GFP*$^{+}$-hematopoietic cells and *flk-GFP*$^{+}$-endothelial cells from WT and *chd8*$^{-/-}$ embryos, and in adult kidney marrow from 6-month-old WT and *chd8*$^{-/-}$ zebrafish. Data are expressed as mean ± SD from three independent experiments (two-tailed Student's t-test, *p < 0.05, **p < 0.01, $^{\#}$p < 0.001, n.s., not significant).

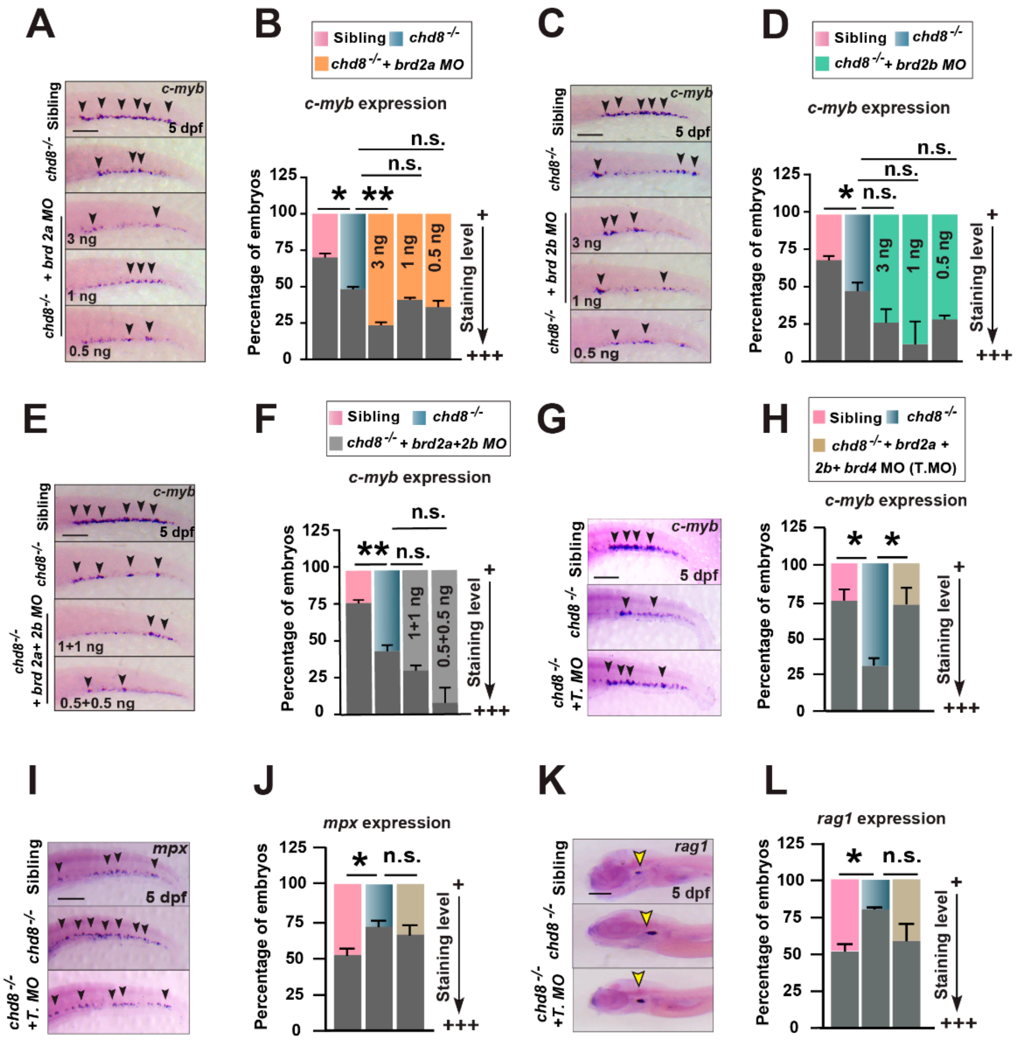

Figure S5. Knockdown of *brd2a*/*brd2b* failed to rescue HSPC defects in *chd8*$^{-/-}$ embryos.

(A, C, E) WISH analysis for *c-myb* in WT siblings, *chd8*$^{-/-}$ embryos and *chd8*$^{-/-}$ embryos following injection with morpholinos (MOs) against *brd2a*, *brd2b*, or a combination of both. MO concentrations are indicated. (B, D, F) Quantification of WISH analysis in (A, C, E). (G, I, K) WISH analysis for *c-myb, mpx,* and *rag1* in WT siblings, *chd8*$^{-/-}$ embryos and *chd8*$^{-/-}$ embryos following injection with a triple morpholino combination against *brd2a, brd2b,* and *brd4*. (H, J, L) Quantification of WISH analysis in (G, I, K). Data are expressed as mean ± SD from three independent experiments (two-tailed Student's t-test, n=10-20 embryos per group, each experiment, $**p < 0.01$, n.s., not significant). ng, nanogram; T, triple; AKM, adult kidney marrow. Scale bars, 100 μm.

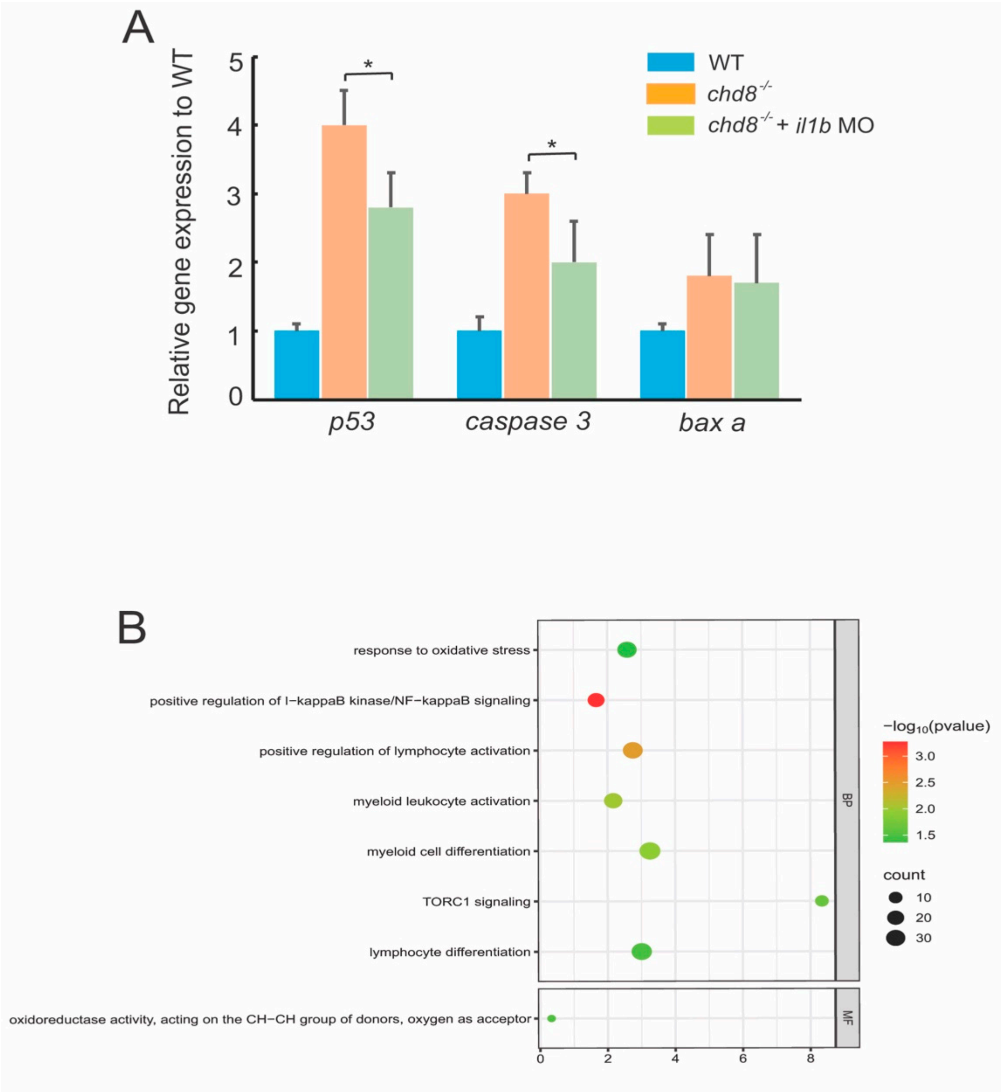

Figure S6. Knockdown *il1b* partially reduces apoptosis in *chd8*$^{-/-}$ zebrafish embryos and myeloid and lymphoid differentiation signaling are increased in mouse *Chd8*$^{\Delta/\Delta}$ HSCs.

(A) RT-qPCR analysis of *p53*, *caspase 3*, and *baxa* expression in WT embryos, *chd8*$^{-/-}$ embryos and *chd8*$^{-/-}$ embryos following injection with MO against *il1b*. (B) Gene ontology (GO) analysis of differentially expressed genes revealed that myeloid and lymphoid differentiation signaling are enriched in upregulated genes in mouse HSCs (*Chd8*$^{\Delta/\Delta}$ vs control). RNA-seq data are downloaded from Nita et al [19].
